# 46, XY disorder of sex development (DSD) complicated by a serous borderline tumor of the ovary: a case report and review of the literature

**DOI:** 10.1186/s13000-020-01010-1

**Published:** 2020-07-23

**Authors:** Jiangying Zhao, Jiao Peng, Sisi He, Jia Yang, Xiaojun Pang

**Affiliations:** 1 Department of Pathology, Mianyang Hospital of T.C.M, Mianyang, Sichuan 621000 P.R. China; 2 Department of Gynecology, Mianyang Hospital of T.C.M, Mianyang, Sichuan 621000 P.R. China

**Keywords:** Disorder of sex development, Gonadal tumors, Serous borderline tumor, Cryptorchidism, Streak ovary

## Abstract

**Background:**

Patients with 46, XY disorder of sex development (DSD) are predisposed to the development of gonadal tumors, particularly germ cell tumors and gonadoblastoma. However, to the best of our knowledge, there are no publications in the existing literature that refer to the coexistence of 46, XY DSD and serous tumors in the ovary.

**Case presentation:**

Here, we report the case of a 23-year-old female (social gender) patient with 46, XY DSD presenting with primary amenorrhea. Imageology revealed a huge mass in the left adnexa. Subsequent pathological analysis revealed a serous borderline tumor of the ovary.

**Conclusion:**

Gonadal tumors of patients with 46, XY DSD are not necessarily malignant tumors and can coexist with borderline tumors with primitive corded gonads. The coexistence of DSD and serous borderline tumors is rare. Clearly, an early and accurate diagnosis plays an important role in the treatment of these patients. Although there may not be a clear correlation between the two lesions, it is vital that we specifically analyze the mechanisms involved so that we can determine whether patients with DSD are associated with an increase of developing serous borderline tumors of the gonad.

## Introduction

Disorder of sex development (DSD) is a congenital disease that is characterized by inconsistencies in the gonadal anatomy, gonadal phenotype and chromosomal karyotype. The incidence of this disorder is approximately 1/5000 to 1/4500 [1]. DSD can be divided into three types according to the chromosomal karyotype: DSD caused by abnormalities of the sex chromosomes; 46, XY DSD; and 46, XX DSD. DSD caused by sex chromosome abnormality is characterized by an abnormal structure and an abnormal number of sex chromosomes. In contrast, cases of 46, XY or 46, XX DSD exhibit a normal structure and a normal number of sex chromosomes, but show inconsistencies between the karyotype of the sex chromosomes and the anatomical structure or phenotype of the gonad. Some studies have shown that the incidence of gonadal tumors is significantly higher in patients with DSD carrying the Y chromosome than in patients with other types of DSD and can reach 50% in patients with 46, XY DSD [2]. This may be related to abnormalities in the expression of SRY, SOX9, SF1, DAX1, WT1 and other genes located on the Y chromosome [3]. Gonadal tumors in these patients are mainly germ cell-sex cord stromal tumors and malignant germ cell tumors, including gonadoblastomas, asexual cell tumors, seminomas and Sertoli cell tumors [4]. The surgical resection of abnormal gonadal tissue is the best way to prevent gonadal tumors in patients with DSD; there are no other forms of treatment for this condition. These patients require regular clinical observation and post-surgical follow-up.

DSD is extremely difficult to diagnose before puberty if there are no abnormalities in the external genitalia or sexual characteristics. Patients with 46, XY DSD with female phenotypes often exhibit primary amenorrhea and sexual problems. Ultrasound or imaging examination often reveal an abnormal gonadal structure or location, including streak ovary, an abnormal or missing uterus and abnormal fallopian tubes; sometimes, these are conditions complicated by cryptorchidism. In such cases, it is necessary to surgically remove the affected tissue for pathological examination and then make a definitive diagnosis according to pathological data; however, even this can be challenging for the pathologist. The provision of an accurate pathological diagnosis is critical for deciding how to treat a patient and estimating the prognosis. To the best of our knowledge, there have been no previous reports relating to DSD complicated by serous borderline tumors of the ovary. Herein, we report the first case of a patient with 46, XY DSD complicated by a serous borderline tumor of the ovary.

## Case presentation

A 23-year-old Chinese female (social gender) presented to our hospital to receive treatment for primary amenorrhea. Physical examination showed normal breast development, an immature vulva, development of the vulva in infancy, no pubic hair, a blind end in the deep part of the vagina, no penile or testicular tissue, and no hypospadias. With the consent of the patient, we performed computed tomography (CT); this revealed huge cystic lesions in the pelvic cavity in the right lower abdomen. Many nodules were present in the inner wall of the cyst and a cord shadow was evident on the left edge of the mass. We were unable to visualize the uterus or bilateral fallopian tubes. Ultrasonography revealed a huge mass in the left adnexal region; we were unable to detect a normal uterine sonogram in the anatomical area of the uterus. The area close to the right adnexal region presented with a 3.0 × 1.3 cm^2^ striped weak echo area by ultrasound; we considered this to be the ‘primordial uterus’.

**Fig. 1:**
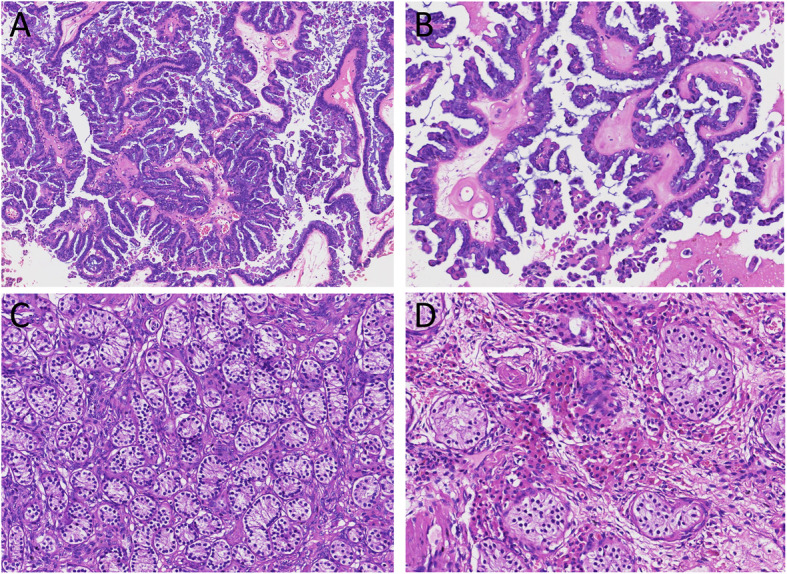
Histological features (hematoxylin and eosin stain). **a** Microscopic observations revealed a multi-stage branching structure and a large number of irregular papillations. (H&E × 100). **b** These papillations branched gradually from largest to smallest and were covered with a cubic columnar epithelium and a ciliated, eosinophilic, cytoplasm. (H&E × 200). **c** These tubules were composed of multiple layers of cells that exhibited cytoplasm that stained in either a rich or light manner with hematoxylin and eosin. These structures were Considered to be Sertoli cells in seminiferous tubules. (H&E × 100). **d** There are eosinophils around these seminiferous tubules; These structures were Considered to be Leydig cells. (H&E × 200)

**Fig. 2:**
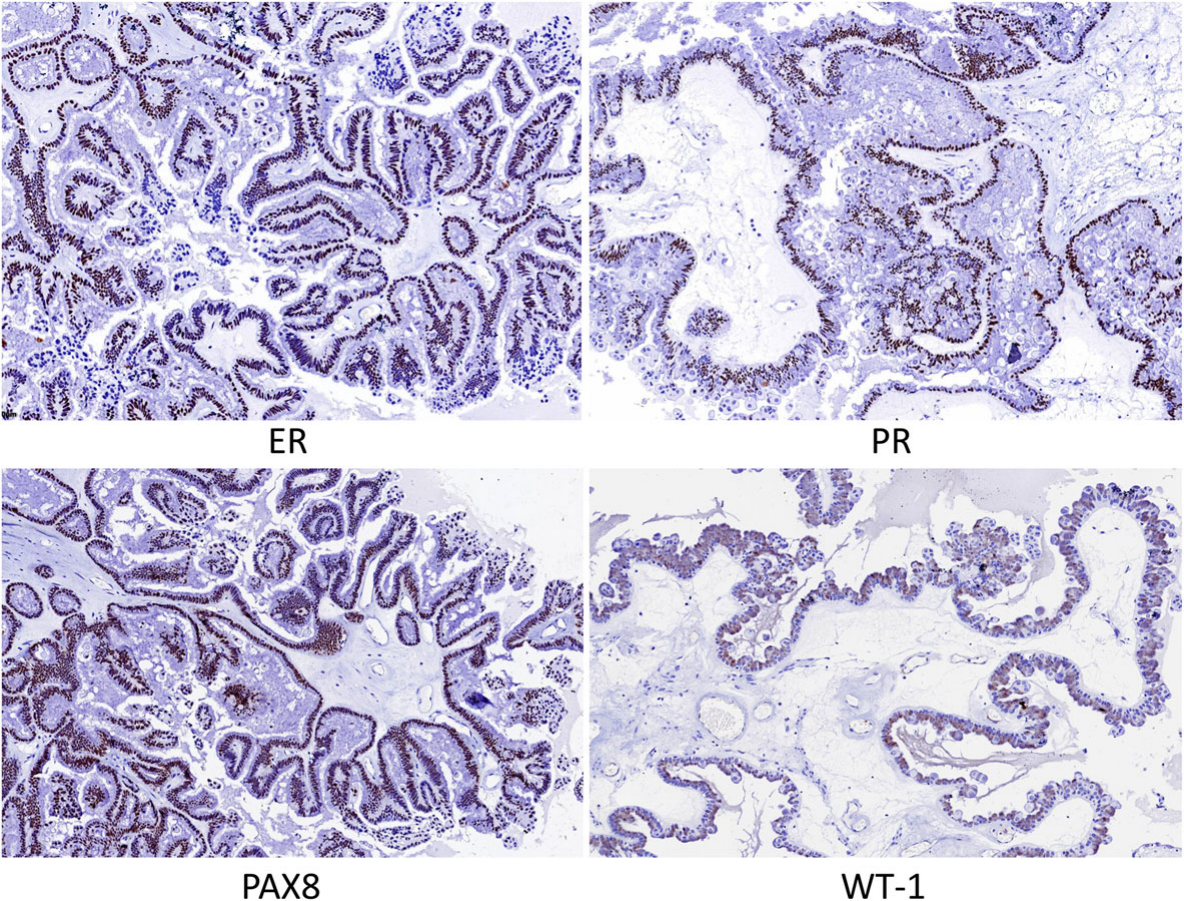
Immunohistochemical staining showed that the tumor cells expressed high levels of estrogen receptor (ER), progesterone receptor (PR), WT1, PAX8, CK7 and EMA; P53 was expressed but wild type and P16 was only expressed at low levels. (IHC × 100)

Following surgical resection, we examined the gross morphology of the specimens. The mass was cystic, and the structure of the mass was papillary. Microscopic observations revealed a multi-stage branching structure and a large number of irregular papillations (Fig. 1a). These papillations branched gradually from largest to smallest and were covered with a cubic columnar epithelium and a ciliated, eosinophilic, cytoplasm (Fig. 1b). Abnormal streak ovarian tissue was evident around the mass. A large number of fibrous tissues were also evident in the specimen, although there were no mature follicles. Immunohistochemical staining showed that the tumor cells expressed high levels of estrogen receptor (ER), progesterone receptor (PR), WT1, PAX8, CK7 and EMA; P53 was expressed but wild type and P16 was only expressed at low levels (Fig. 2). The morphological characteristics evident on hematoxylin and eosin staining, along with the immunohistochemical results, were indicative of a serous borderline tumor of the ovary.

**Fig. 3:**
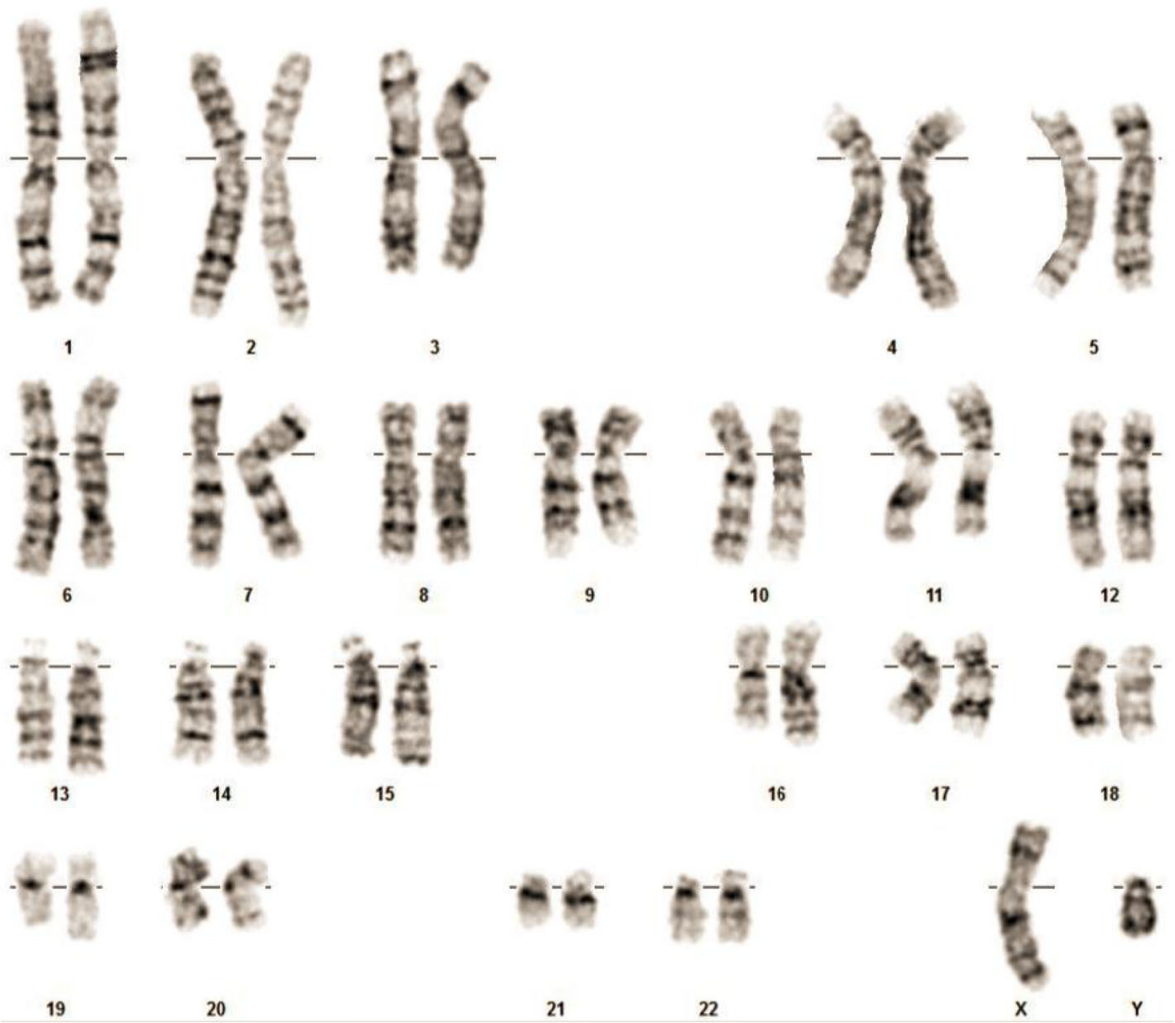
The sex chromosomes revealed that the patient was 46, XY

The tissue that we considered as the “primordial uterus” by ultrasound was located in an area close to the right adnexal region. Anatomical analysis revealed that this tissue was atrophic, cord-like, and smooth; it was also surrounded by a capsule and was devoid of ligaments and fallopian tubes. We were surprised by the lack of a uterine cavity, endometrium, or muscle wall, although the yellowish, spongy, soft tissue appeared to be denatured and atrophic tubular tissue. These tubules were composed of multiple layers of cells that exhibited cytoplasm that stained in either a rich or light manner with hematoxylin and eosin. We believe that these structures were Sertoli cells in seminiferous tubules (Fig. 1c). We also observed eosinophils around these seminiferous tubules; we believe that these structures were Leydig cells. Consequently, we believe that this structure was actually an immature testis (Fig. 1d). Based on microscopic observations of the tissue, we considered that this was not a primordial uterus; rather, this was a case of cryptorchidism. Further examination of the sex chromosomes revealed that the patient was 46, XY (Fig. 3); her disorder was therefore diagnosed definitively as 46, XY DSD. The patient had testes, which were able to secrete Mullerian tube inhibitor, thus inhibiting the development of the uterus and fallopian tubes. The patient had also undergone normal breast development and had a female appearance, although her vagina had a blind end and the external genitalia appeared female without pubic hair. The development of secondary sexual characteristics was abnormal; there was no uterus, no fallopian tubes, and no hypospadias. Although sex hormone tests revealed high levels of testosterone, there was no obvious development of male external genitalia. Therefore, the patient was diagnosed with complete androgen insensitivity syndrome complicated by a serous borderline tumor of the ovary. After 6-months of follow-up, the patient was in good condition.

## Discussion

DSD presents with no typical signs during imaging assessments. Usually abnormal lesions are found inadvertently instead of normal gonadal tissue. These abnormal lesions may be considered as neoplastic lesions. If the patient’s social gender and external genitalia develop normally, it is difficult to consider the likelihood of ectopic gonads or dysgenesis on the basis of imaging findings. It is not difficult to detect ovarian tumors on imaging findings, although pathological examination will be needed to determine the specific type of tumor. During the course of disease analysis, it is difficult for clinicians to make an accurate diagnosis based on imaging or ultrasonography findings.

According to a review of the available evidence relating to the histological origin of ovarian tumorigenesis, we believe that a serous epithelium arises in the ovary by way of the development of a special mesothelium on the ovarian surface, possibly via Mullerian epithelial metaplastic changes during the transition to a tubal epithelium [5]; however, the specific mechanisms involved need to be elucidated. Although a serous borderline tumor of the ovary is not malignant, there is a risk of these tumors transforming into low-grade serous cancer. A streak ovary lacks normal embryonic development, contains a significant extent of fibrous tissue and has some characteristics of a primitive ovarian structure. This is because gonadal tumors in patients with 46, XY DSD are mainly gonadoblastoma and germ cell tumors, and contain no epithelial ovarian tumors. Therefore, we considered that our present case was rare and should be reported to the wider clinical community. We strongly believe that the diagnosis of DSD and gonadal tumors should rely on specific pathological examination and the detection of sex chromosomes. An abnormal Y chromosome can exert complex effects, including a disturbance in hormonal levels, the inactivation of self-regulation, and the promotion of clonal growth in tumor cells. Certain factors, such as the expression of different gene fragments in the Y chromosome, along with individual gene rearrangements and mutation, can lead to the development of abnormal gonadal tumors. Although many tumor-related genes have been found in the Y chromosome, the mechanisms underlying the effects of the Y chromosome on different types of gonadal tumor in patients with DSD have yet to be fully elucidated [6, 7].

Surgical resection of dysplastic gonads is currently the best way to prevent tumorigenesis or the malignant transformation of benign tumors. However, gonadectomy can be postponed until after puberty to ensure that puberty progresses and to avoid the influence of hormone replacement therapy on the patient’s body [8]. In our case, the streak ovary formed the basis for a serous borderline tumor; ovariectomy was effectively able to prevent exacerbation of the tumor. However, we must also consider that contralateral cryptorchidism is also a hidden danger for tumorigenesis. Consequently, resection of the cryptorchidism can significantly reduce the risk of tumorigenesis. There are many types of DSDs; the diagnosis and classification of these conditions, as well as chromosomal detection, play an important role in determining prognosis and in the management of treatment. Such patients are very difficult to diagnose from a pathological point of view.

According to the available evidence relating to the mechanisms underlying serous borderline tumors of the ovary, it has not yet been established how 46, XY DSD promotes the development of this particular type of tumor. In our case, it was also difficult to determine whether the tumor was associated with the diagnosis of 46, XY DSD. Indeed, while DSD and serous borderline tumors may be independent and non-associated lesions, our present case presented with both conditions. Although, cases of 46, XY DSD are rare, it is very evident from the literature that cases of 46, XY DSD with serous borderline tumors of the ovary, are even rarer. Due to the lack of such cases, little is known about the association between the two conditions, particularly from a mechanistic point of view. With regards to our particular patient, the important point to note is that gonadal tumors in patients with DSD may not necessarily be malignant but can be borderline. It is vital that we investigate the relationship between DSD and gonadal tumors in much more detail with as many cases as possible. In such work, it is important that we include molecular genetic investigations.

## Conclusion

In conclusion, it is rare to encounter cases of 46, XY DSD with a serous borderline tumor of the ovary. Although the two diseases can occur independently and have no specific association with each other, their simultaneous occurrence in the patient reported herein, deserves further attention. Our patient had no uterus, and one ovary had been replaced by an undescended testicle. In addition, epithelial tumors were evident in the streak ovary. All of these conditions are difficult to detect prior to surgery. However, in the event of primary amenorrhea and abnormal development of the vulva, we should consider the possibility of DSD. The gonadal tumors of patients with DSD are not all malignant but can represent epithelial borderline tumors. In patients with 46, XY DSD with gonadal tumors, the likelihood of borderline epithelial tumors should also be taken into account; this can be confirmed by pathological examination. Further studies, however, are still needed to identify whether there is a specific relationship between 46, XY DSD and the development of serous borderline tumors of the gonad.

## Supplementary information

**Additional file 1.**

## Data Availability

Data sharing is not applicable to this article as no datasets were generated or analysed during the current study.

## Abbreviations

DSD: Disorder of sex development
CT: Computed tomography
ER: Estrogen receptor
PR: Progesterone receptor
WT1: Wilms’ tumor 1
PAX8: Paired box 8
CK7: Cytokeratin 7
EMA: Epithelial membrane antigen
