# Supplementary figures and images for: 46, XY disorder of sex development (DSD) complicated by a serous borderline tumor of the ovary: a case report and review of the literature
Source: Diagn Pathol. 2020 Jul 23;15:93. doi: 10.1186/s13000-020-01010-1 (PMC7376885; doi:10.1186/s13000-020-01010-1)

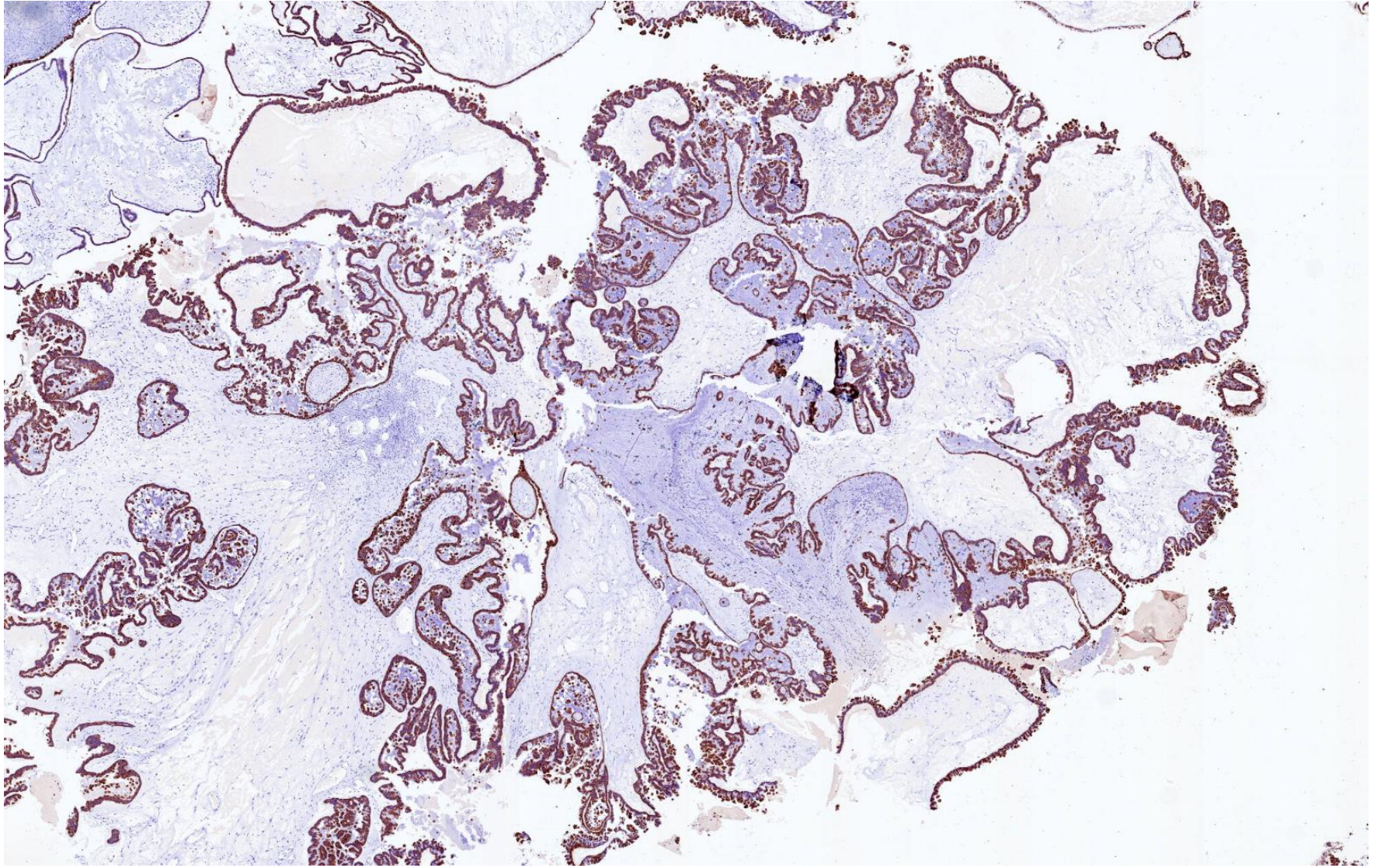

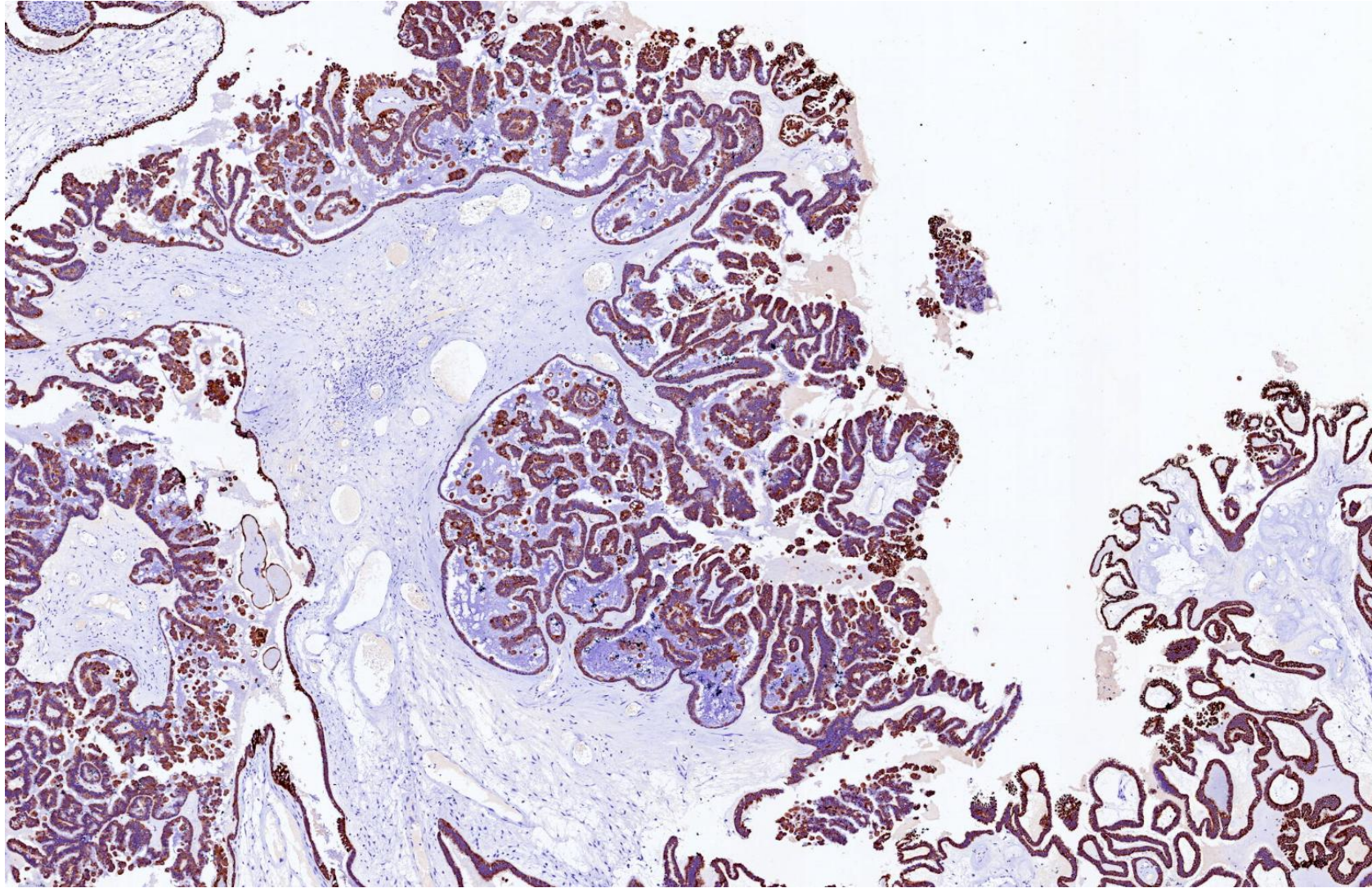

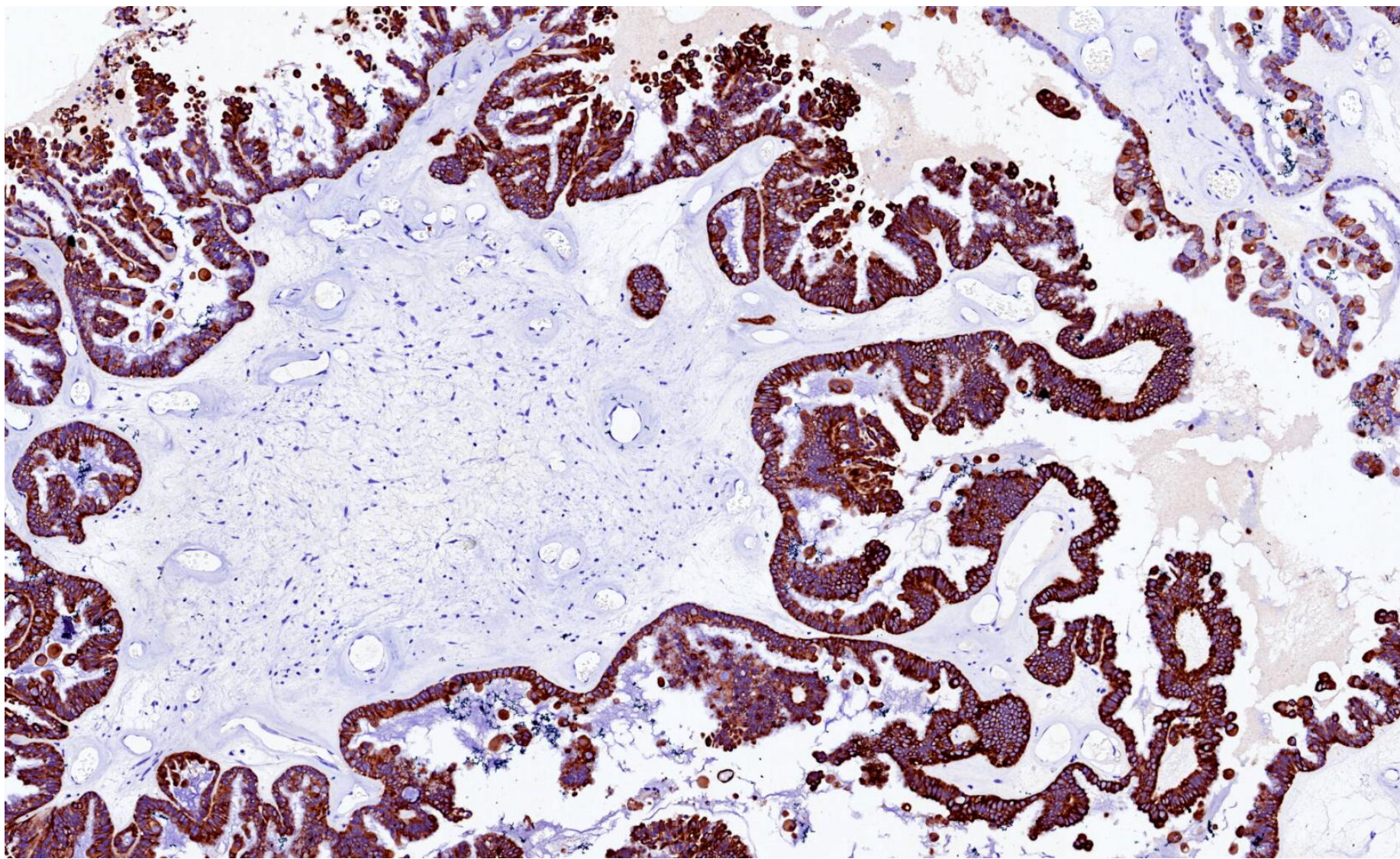

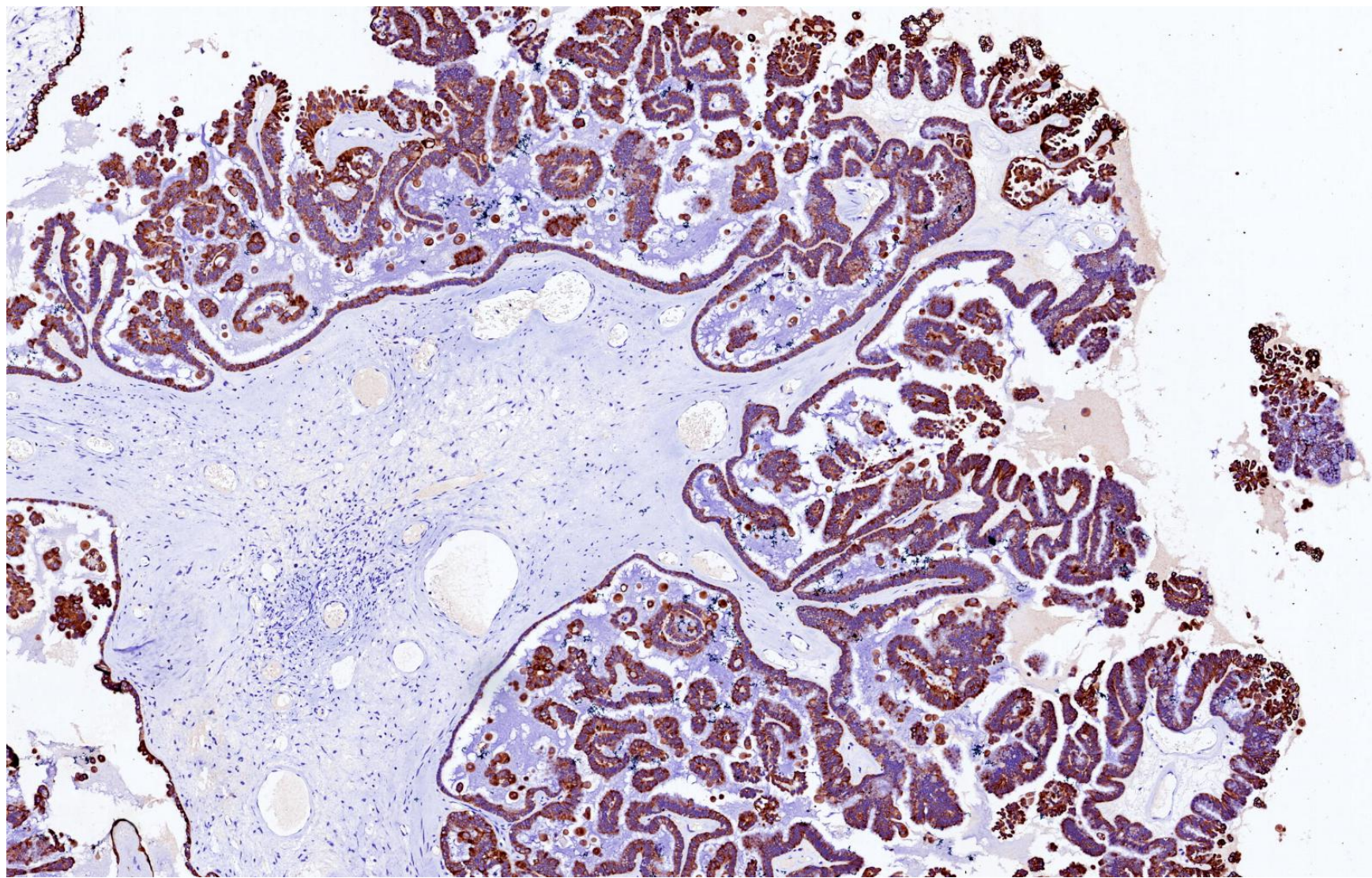

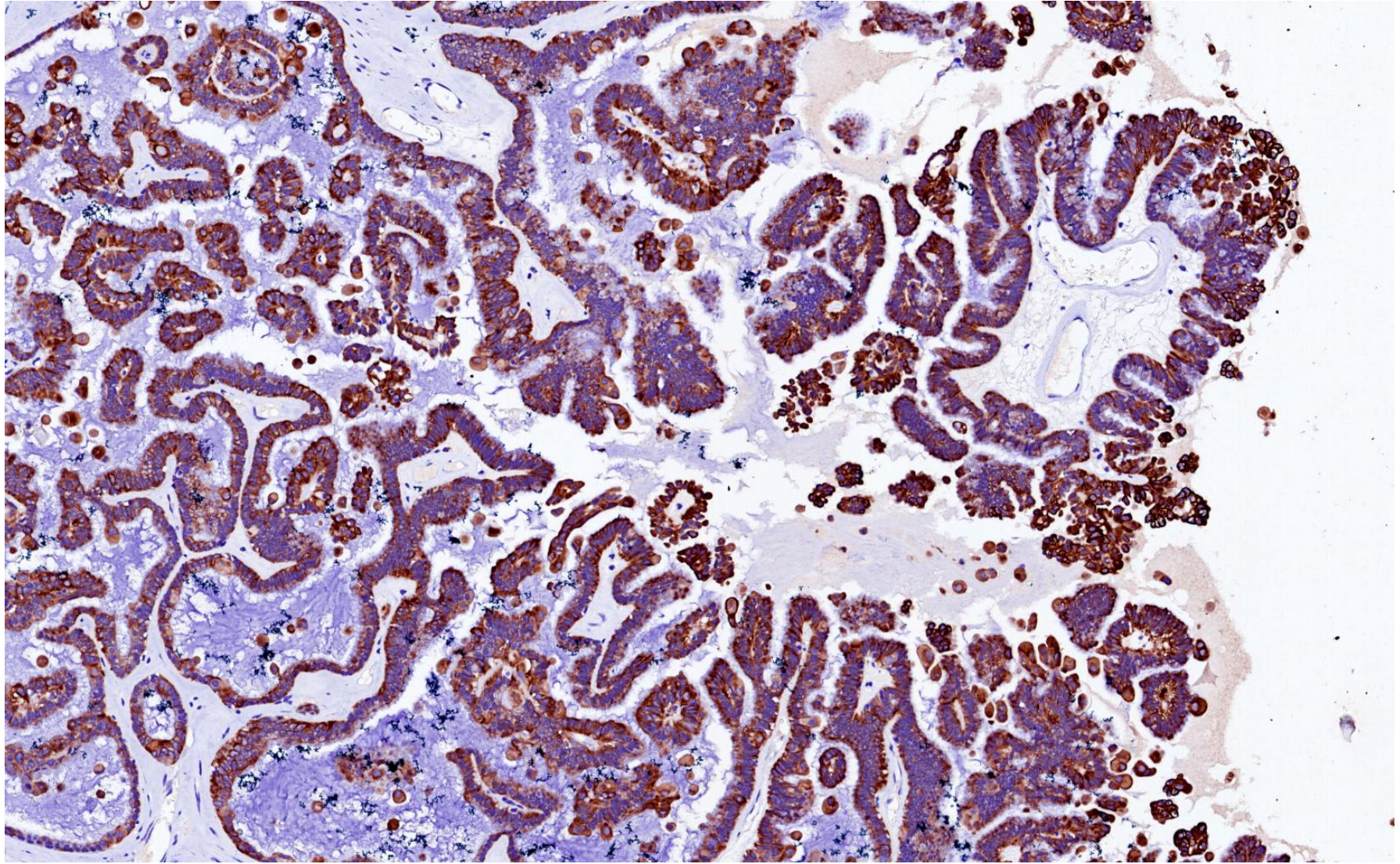

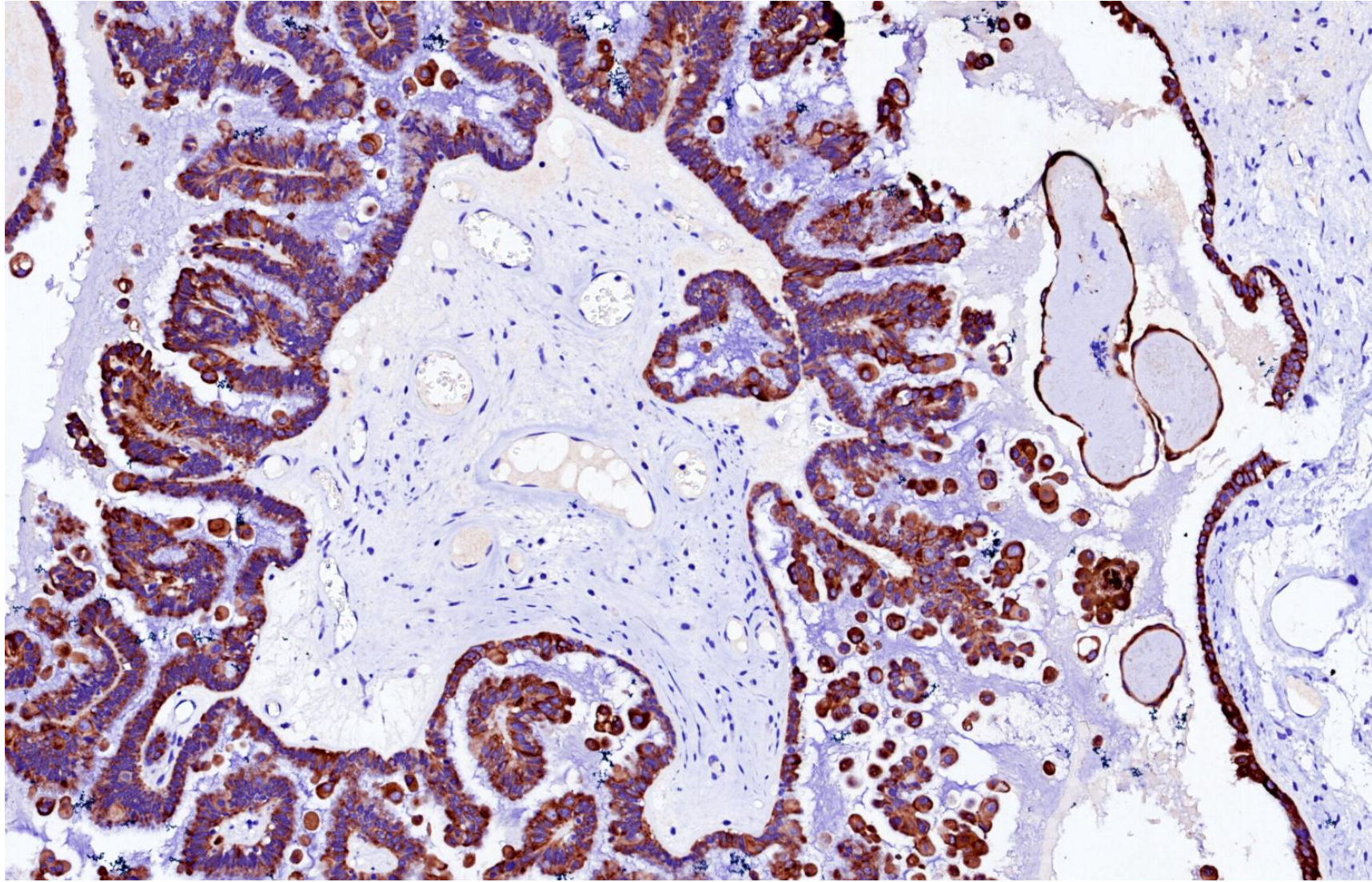

Supplement: Supplementary file 1 — Additional file 1. [file 13000_2020_1010_MOESM1_ESM.pdf]
